# A Multi-parametric MRI-Based Radiomics Signature and a Practical ML Model for Stratifying Glioblastoma Patients Based on Survival Toward Precision Oncology

**DOI:** 10.3389/fncom.2019.00058

**Published:** 2019-08-27

**Authors:** Alexander F. I. Osman

**Affiliations:** Department of Medical Physics, Al-Neelain University, Khartoum, Sudan

**Keywords:** glioblastoma multiforme, MRI, radiomics analysis, patient's survival prediction, machine learning, precision oncology

## Abstract

**Purpose:** Predicting patients' survival outcomes is recognized of key importance to clinicians in oncology toward determining an ideal course of treatment and patient management. This study applies radiomics analysis on pre-operative multi-parametric MRI of patients with glioblastoma from multiple institutions to identify a signature and a practical machine learning model for stratifying patients into groups based on overall survival.

**Methods:** This study included 163 patients' data with glioblastoma, collected by BRATS 2018 Challenge from multiple institutions. In this proposed method, a set of 147 radiomics image features were extracted locally from three tumor sub-regions on standardized pre-operative multi-parametric MR images. LASSO regression was applied for identifying an informative subset of chosen features whereas a Cox model used to obtain the coefficients of those selected features. Then, a radiomics signature model of 9 features was constructed on the discovery set and it performance was evaluated for patients stratification into short- (<10 months), medium- (10–15 months), and long-survivors (>15 months) groups. Eight ML classification models, trained and then cross-validated, were tested to assess a range of survival prediction performance as a function of the choice of features.

**Results:** The proposed mpMRI radiomics signature model had a statistically significant association with survival (*P* < 0.001) in the training set, but was not confirmed (*P* = 0.110) in the validation cohort. Its performance in the validation set had a sensitivity of 0.476 (short-), 0.231 (medium-), and 0.600 (long-survivors), and specificity of 0.667 (short-), 0.732 (medium-), and 0.794 (long-survivors). Among the tested ML classifiers, the ensemble learning model's results showed superior performance in predicting the survival classes, with an overall accuracy of 57.8% and AUC of 0.81 for short-, 0.47 for medium-, and 0.72 for long-survivors using the LASSO selected features combined with clinical factors.

**Conclusion:** A derived GLCM feature, representing intra-tumoral inhomogeneity, was found to have a high association with survival. Clinical factors, when added to the radiomics image features, boosted the performance of the ML classification model in predicting individual glioblastoma patient's survival prognosis, which can improve prognostic quality a further step toward precision oncology.

## Introduction And Related Works

### Introduction

Glioblastoma multiforme (GBM) is the most aggressive and highly invasive high-grade glioma tumors with poor prognosis (Holland, 2001). The median survival rate of GBM patients is about 2 years or less, and it needs immediate treatment (Ohgaki and Kleihues, 2005; Louis et al., 2007). Surgical resection followed by chemo-radiotherapy is the current standard treatment of the glioblastoma multiforme tumors (Van Meir et al., 2010; Aum et al., 2014). Predicting a patient's survival outcome is recognized as key importance to clinicians in oncology toward determining an ideal course of treatment and patient management. In which, the treating physician (oncologist) may decide if more aggressive or additional treatment has to be considered for treating patients with poor survival prognosis (Zhang et al., 2017).

Multi-parametric magnetic resonance imaging (mpMRI) sequences commonly provide more clinical information to characterize glioblastoma multiforme tumors than other imaging modalities. Here, “multi-parametric” is refereed to multiple image standardization parameters. This imaging information could be quantitatively extracted as features and linking these tumor phenotype features to clinical variables of interest (e.g., survival time, recurrence, adverse events, or late complications). The mentioned concept is referred to as radiomics. The idea of radiomics has recently emerged from the field of oncology. Radiomics has the potential for enabling improved clinical decision-making (Gillies et al., 2016). This approach has advantages of being non-invasive, fast and low in cost. Radiomics has been used in oncology for tumors' diagnosis, treatment planning/execution, treatment response and prognosis, and underlying genomic patterns in various forms of cancer (Liu et al., 2018a). In which, individual patients could be stratified into subtypes based on radiomics biomarkers that hold information about cancer traits that reflect the patient's prognosis. As a result, radiomics could have an effective application in precision oncology by predicting individual patients' treatment outcome.

The definition of precision medicine, according to the National Institute of Health (NIH), is “an emerging approach for disease treatment and prevention that takes into account individual variability in genes, environment, and lifestyle for each person” (Subramaniam, 2017). This concept will let clinicians and researchers provide predictions with higher accuracy for which treatment and prevention plans for a particular disease will suit in which groups of people (Subramaniam, 2017). The newly introduced idea of precision medicine is in contrast to the existing practical therapy paradigm of a “one-size-fits-all” attitude, in which disease treatment and prevention plans are developed for the “average” patient, with less consideration for the differences between individuals (Subramaniam, 2017). There are some limitations in fully implementing precision medicine for radiomics e.g., reproducibility and quantitative information, standardization in image acquisition, and structured reporting.

### The Related Works

Many studies have been conducted identifying tumor phenotypical radiomics signature or/and developing practical machine learning (ML) models for glioblastoma patients stratification based on survival on pre-operative multi-parametric MRI sequences from single or multiple institutions. Recognizing patients who would/wouldn't benefit from standard treatment as well as identifying patients who need more aggressive treatment at the time of diagnosis is essential toward management of glioblastoma through personalized medicine. In this section, the author included some works of the most relevant ones recently published in this field. Macyszyn et al. (2016) used image analysis and ML models to establish imaging patterns that are predictive of overall survival (OS) and molecular subtype using preoperative mpMRIs sequences of patients with GBM. The developed system achieved an overall accuracy of 80% in stratifying patients into long-, medium-, and short-term survivors in the prospective cohort from a single institution. Prasanna et al. (2017) studied texture features analysis to assess the efficacy of peritumoral brain zone features from pre-operative MRI in predicting GBM patient survival into long- (>18 months) vs. short-term (<7 months). The study findings identified a subset of 10 features proven to be predictive of long- vs. short-term survival as compared to known clinical factors. Ingrisch et al. (2017) investigated whether radiomics analysis with random survival forests can predict overall survival from MRI scans of newly diagnosed glioblastoma patients. Their results demonstrated that low predicted individual mortality proven to be a favorable prognostic factor for OS, it also indicated that the MRI contains prognostic information, which can be accessed by radiomics analysis.

Most recently, Chaddad et al. (2018) proposed multiscale texture features for predicting GBM patients' progression-free survival and overall survival on T1 and T2-FLAIR MRIs using the random forest. The study results showed that the identified seven-feature set, when combined with clinical factors, improved the model performance yielding an AUC value of 85.54% for OS predictions. Kickingereder et al. (2018) investigated the impact of mpMRI radiomics features for predicting patients' survival in newly diagnosed GBM patients before treatment. The study results revealed that a constructed eight-feature radiomics signature increased the prediction accuracy for OS further than the alternative approaches. Sanghani et al. (2018) studied survival prediction of glioblastoma patients for two-class (short- vs. long-term) and three-class (short-, medium-, and long-term) survival groups using Support Vector Machines (SVMs). The results showed a prediction accuracy of 98.7 and 88.95% for two-class and three-class OS group, respectively. Chen et al. (2019) studied developing a post-T1-weighted MRI-based prognostic radiomics classification system in GBM patients to assess if it could allow stratifying patients into a low- or high-risk group. Their results showed that the developed system classified patients' survival with improved performance with AUC of 0.851 for 12-month survival, compared to conventional risk models.

The majority of those studies have performed on single-institution data, and also survival grouping was designed for two-class rather than three-class approach. Besides, implementing a particular feature selection method and testing various machine learning classification models allow greater flexibility for exploring distinct methods. The purpose of this work is to quantitatively study the radiomics features from pre-operative multi-parametric MRI of the *de novo* glioblastoma tumor on multi-institutional datasets. Then, to apply radiomics analysis on mpMRI to identify a signature and a practical machine learning model to stratify patients into short-, medium, and long-survivors groups. For machine learning, different models were tested to assess a range of performance as a function of the choice of features.

## Materials and Methods

### Patients Data Sets

**Figure 1:**
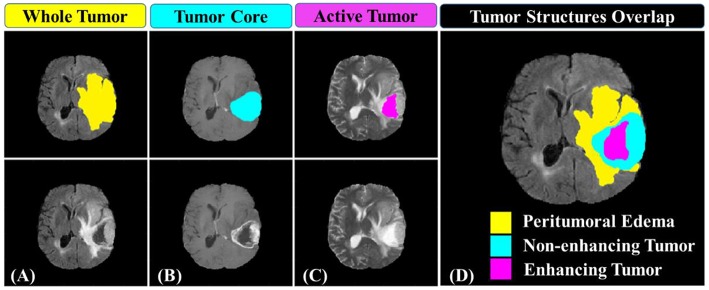
Glioblastoma multiforme sub-regions segmentation masks generated by experts annotated in the different MRI sequences. **(A)** the whole tumor (yellow) visible in T2-FLAIR, **(B)** the tumor core (light blue) visible in T2, and **(C)** the active tumor structures (purple) visible in T1-Gd. Combination of three segmentation labels overlaid on T2-FLAIR MRI producing **(D)** the final labels of the tumor sub-structures: peritumoral edema [ED] (yellow), non-enhancing solid core tumor [NET] (light blue), necrosis [NCR], and enhancing tumor core (purple).

The study involved a cohort of 163 patients diagnosed with primary *de novo* GBM and pathologically confirmed. The patients' imaging data sets and clinical information data were collected from multiple (*n* = 5) institutions and provided as “training data set” for Multimodal Brain Tumor Segmentation (BRATS) 2018 Challenge (Menze et al., 2015; Bakas et al., 2017a,b,c). For each patient, the imaging data set consisted of four sequences of pre-operative multi-parametric MRIs along with the patient's clinical information. The imaging data sets were acquired during regular clinical routine using various scanners, and different scanning protocols. An individual patient's imaging data set included T1-weighted (T1), T1-weighted with post-contrast/gadolinium (T1-Gd), T2-weighted (T2), and T2-weighted fluid-attenuated inversion recovery (T2-FLAIR) MRI sequences. Besides, “ground truth” segmentation masks of three tumor sub-structures provided as follow: the complete tumor extent also referred to as the “whole tumor” (WT), tumor core (TC), and the active tumor (AT) and the non-enhancing/necrotic tumor region (Figure 1). The clinical data were composed of the patient's age, patient's overall survival, and tumor's resection status information. The demographic and clinical characteristics data of the glioblastoma patients in the discovery, validation, and in the combined cohorts, were presented in Table 1.

**Table 1:** Demographic and clinical characteristics data of GBM patients in discovery, validation, and combined sets.

**Characteristic**	**Discovery**	**Validation**	**Combined**
***Patients demographic***			
**No. of patients**			
Patient distribution	109 (67%)	54 (33%)	163
-CBICA UPenn	–	–	85 (52%)
-TCIA	–	–	76 (47%)
- MGH, HU, DU, and BU	–	–	2 (1%)
***Imaging data***			
- Data set of T1, T1-Gd, T2, and T2-FLAIR MRI sequences with tumor sub-structures “ground truth” segmentation labels	–	–	163
***Clinical information***			
**Age (years) (*****P*** **=** **0.368)^†^**			
- Range	18.97–84.84	33.88–85.76	18.97–85.76
- Mean	59.73	61.55	60.33
- Median	60.94	62.36	61.17
−1 Standard deviation	12.23	11.81	12.03
**Overall survival (days) (*****P*** **=** **0.934)^†^**			
- Range	5–1767	22–1731	5–1767
- Mean	421.37	426.18	422.96
- Median	362.00	364.50	362.00
-1 Standard deviation	350.00	352.31	349.67
- Short-term survivors [ <10 months]	44	21	65 (40%)
- Medium-term survivors [10–15 months]	28	14	42 (26%)
- Long-term survivors [>15 months]	37	19	56 (34%)
**Resection status (*****P*** **=** **0.474)^†^**			
- Gross total resection	36	23	59 (36%)
- Subtotal resection	19	5	24 (15%)
-Missing information	54	26	80 (49%)

*CBICA UPenn, Center for Biomedical Image Computing and Analytics at the University of Pennsylvania; TCIA, The Cancer Imaging Archive; BU, Bern University; DU, Debrecen University; HU, Heidelberg University; MGH, Massachusetts General Hospital*.

† *Data in parentheses are P-value*.

The patient data sets were categorized into discovery/training and validation cohorts. In which, the survival data were sorted in order hence after every two consecutive values the third one was chosen for validation and added to the validation data set while the remained ones were considered as the discovery data set. This distribution of overall survival data across the discovery and validation data sets ensure a balanced appearance of the whole OS values range (from short, through a medium, to long-survivors) in both cohorts. The patients' survival data were categorized into long- (>15 months), medium- (between 10 and 15 months), and short-term survivors (<10 months) groups. The reason behind choosing these thresholds can be found with a detailed explanation by referring to this BRATS paper (Bakas et al., 2019).

### Annotation of Tumor Structures

The extracted radiomics features may suffer from the robustness due to variations in the delineated tumor structures. Consequently, a decision was made to use the provided “ground truth” segmentation masks which were manually generated by experts, rather than using the author's developed automated segmentation system (Osman, 2018) which was still under further improving. The tumor sub-structures delineation was performed by experts (one to four raters) using the multi-parametric MR images following a specific given annotation protocol. The experts' annotations were further revised by an experienced board-certified neuroradiologist to minimize inter- and intra-raters variations (Menze et al., 2015; Bakas et al., 2019). Three tumor sub-structures were delineated on the imaging data namely; the complete tumor extent also referred to as the “whole tumor,” the tumor core, and the active tumor and the non-enhancing/necrotic tumor region structures (illustrated in Figure 1). The protocol used for annotating the tumor structures was described in detail in those two BRATS papers (Menze et al., 2015; Bakas et al., 2019).

### Image Preprocessing

**Figure 2:**
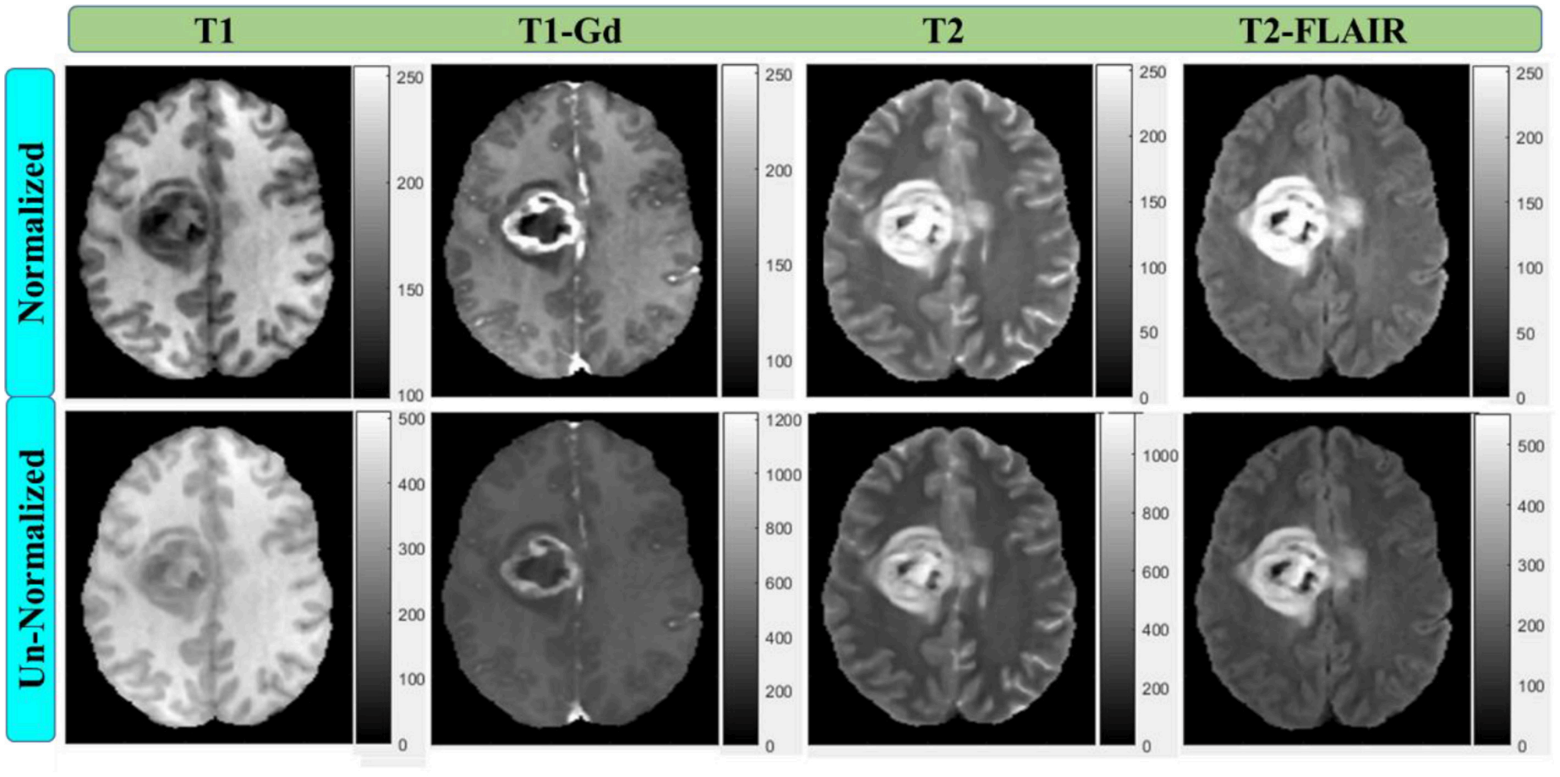
Multi-parametric MRI sequences before and after intensity normalization with 256 scale (8-bit depth). All normalized images have the same scale.

The multi-parametric MR images were provided with initial preprocessing. The four mpMRI sequences of each patient were co-registered using T1-Gd image sequence as a reference. The images were also smoothed, interpolated to the same resolution of 1 mm^3^, and skull-stripped. Each imaging sequence was had 240 × 240 pixels and 155 slices acquisition matrices and converted into grayscale. Further preprocessing were performed to standardize the image intensity before performing features extraction. The most commonly used MRI normalization scheme of μ±3σ with 256 intensity bins (Collewet et al., 2004) was applied. MRI intensity rescaling (Figure 2) on the global brain image volume was employed to convert MRI signal intensity values into a standardized intensity range, thus avoiding bias due to heterogeneity. Image intensities were standardized between μ ± 3σ where μ was the mean value of the gray levels inside the region of interest (brain) and σ the standard deviation. The gray level values outside the [μ − 3σ, μ + 3σ] range were truncated to the upper or lower limit value. The given range was then quantized into 8 bits [0, 255]. This standardization method eliminates the dependency on the shift of the mean value and multiplicative change in the image intensity. In contrast, the relative difference between two gray levels is not maintained (Collewet et al., 2004).

### Feature Extraction and Selection

#### Feature Extraction

For each patient, various features were extracted locally from the “whole tumor”, tumor core, and active tumor areas on the T1-Gd, T2, and T2-FLAIR MRI sequences to capture different phenotypic characteristics of the tumor. The features were divided into the following groups:

- Geometry/shape features: which describe the two-dimensional (2D) and 3D shape characteristics of the tumor.
- Intensity features: which describe the first-order statistical distribution of the voxel intensities obtained from a histogram characterizing heterogeneity without giving spatial information within a tumor.
- Gray-Level Co-occurrence Matrix (GLCM) Texture features: which describe the high-order statistical spatial distributions of the voxel intensities characterizing heterogeneity with spatial information within a tumor or region of interest (Haralick et al., 1973; Haralick and Shapiro, 1992).
- Histogram of Oriented Gradients (HOGs) features: which capture local shape information from regions or point locations within an image (Dalal and Triggs, 2005).
- Local Binary Pattern (LBP) features: which encode local texture information that can be used for tasks such as detection and recognition (Ojala et al., 2002).

The normalized volumetric MRI data were used for 2D and 3D features extraction. The 2D features were extracted from a region of interest on a pre-selected image slice. This slice was chosen to correspond to the largest tumor surface area in axial, sagittal, and coronal planes. Then the transverse slice was picked out for extracting the information. Based on the segmentation results [WT on (T2-FLAIR), TC on (T2), or AT on (T1-Gd)], the region enclosing each tumor sub-structure was cropped down on the image. The obtained image was used to extract feature information. A total of 147 multi-parametric MRI radiomics features were extracted/derived for each patient from the segmented tumor sub-structures on the three mpMRI sequences for their capability to characterize the glioblastoma tumor phenotypes. For every sub-region, a set of 48 radiomics features was obtained, resulting in a total of 144 features for the three regions plus 3 additional ones calculated as a joint of the three regions. The features included 14 geometry/shape (plus 3 mixed) features, 14 statistical intensity features, 14 texture (GLCM) features, and 6 local features representing 3 HOG features and 3 LBP features (listed in Table 2). All features were derived using MATLAB 2016b Toolbox (Mathworks, Natick, MA, USA) with Image Processing and Computer Vision Tools.

**Table 2:** A summary of radiomics features extracted from the tumor sub-regions (WT, TC, and AT) in multi-parametric MR images (T1-Gd, T2, and T2-FLAIR).

**Feature classes**	**Feature names**
Sub-regions (*n* = 3)	Whole tumor (WT), tumor core (TC), and active tumor (AT).
Shape features (*n* = 14 + 3^*^)	Volume [tumor, brain], volume ratio [tumor/brain, AT/WT^*^, TC/WT^*^, AT/TC^*^], surface area [tumor convex area, tumor filled area, tumor area, brain area], surface area ratio [tumor to brain], eccentricity, orientation, equivalent diameter, solidity, extent, perimeter.
Intensity features (*n* = 14)	Minimum value, maximum value, median value, mean value, range, variance, moment 2nd-order, moment 3rd-order, entropy, kurtosis, root mean square (RMS), skewness, standard deviation, mean absolute deviation (MAD).
Texture features: GLCM (*n* = 14)	Contrast, correlation, energy, homogeneity, (sum) variance, (sum) average, (mean) variance, (mean) autocorrelation, entropy, (sum) entropy2, (difference) entropy2, (sum) variance2, (difference) variance2, range of all GLCM features.
HOG features (*n* = 3)	Sum HOG, median HOG, standard deviation HOG.
LBP features (*n* = 3)	Sum LBP, mean LBP, standard deviation LBP.

*All features were extracted from a 2D image except those indicated as volumetric features (3D)*.

Unless noted with a strike (*), each feature was individually extracted from the “whole tumor” area on T2-FLAIR MRI, tumor core area on T2 MRI, and active tumor area on T1-Gd MRI.

* *These features were calculated as combined features from joint of WT, TC, and AT sub-structures*.

*Features indicated with (2) were derived from GLCM calculated horizontally (0-degree) and 45-degree rotations*.

#### Feature Selection

Following the feature extraction, a feature selection method is required to lessen the number of features to consider only the significant ones. Feature selection refers to reduction of the number of parameters to avoid overfitting dilemma while improving the generalizability and interpretability of the training-based model. Accordingly, a two-step method was applied to choose the most important features and throughout the less associated ones. *Initially*, the median absolute deviations (MAD) was calculated for the 147 extracted features. None of the features with MAD equal to zero, which considered as non-informative, was observed in the total set to be discarded. After this step, the number of features remained the same. *Then*, least absolute shrinkage and selection operator (LASSO) generalized linear regression (Tibshirani, 1996) was employed for finding a subset of the most relevant features from the initial set. Basically, LASSO executes a penalty on the log partial likelihood (sum of squares) that is equal to the absolute sum of regression coefficients. Cross-validation the deviance is then used to determine the LASSO tuning parameter λ (Hastie et al., 2009). LASSO minimizes the regression coefficients down toward zero while it makes the coefficients exactly zero for irrelevant features (Collewet et al., 2004). The LASSO method has been used extensively in high-dimensional feature selection when the number of variables exceeds the sample size (Heinze et al., 2018) as a case in this study where the number of extracted imaging features (*n* = 147) is higher than the number of patients (*n* = 109) in the discovery set. When the LASSO regression model was applied here, nine features with non-zero coefficients retained from all features' set. To search for an optimal λ, cross-validation with 10-fold was applied, where the final λ value yielded minimum error in cross-validation (**Figure 4**). The selected subset was considered as the final one of the chosen features which will be used to construct the multi-parametric MRI radiomics signature model on the discovery data set (*n* = 109).

### Constructing and Validating a Radiomics Signature

Using the LASSO regression selected imaging features, a multivariate LASSO Cox regression (Cox and Oakes, 1984) was then applied to obtain the coefficients of those chosen features rather than using the LASSO's coefficients. The reason for using LASSO Cox regression, because it enables getting the *p*-value, and interferes with the coefficients (Tibshirani, 1997). Cox regression is a semiparametric method for fitting survival rate estimates to eliminate the effect of confounding features, and to quantify the effect of predictor features. It has been reported that the LASSO Cox regression model is reliable for prediction of patients' survival in glioma (Chaddad et al., 2019a). The selected image features with their corresponding coefficients were used to construct a mpMRI radiomics signature model. At first, a radiomics risk score for each patient was determined by linearly combining these selected features weighed by their respective fitting coefficients (β) (Liu et al., 2018a) as follows:

$$\begin{array}{r} Risk\;score=\;\sum_{i=1}^{n}\beta_{i}\;{.\;feature}_{i}. \end{array}$$

Then, the risk scores obtained for patients in the discovery set were stratified into low-(long-), medium-(medium-), and high-risk (short-survivors), with fixed cutoff points as thresholds. The steps which the author implemented to find these cutoffs were as following: first, the radiomics risk score was calculated for all patients in the discovery set. Their values ranged between (+)4.118 to (–)1.497 for the short-survivors group (high risk), (+)0.945 to (–)2.619 for the medium-survivors group (medium risk), and (+)1.603 to (–)3.211 for the long-survivors group (low risk). Then, the corresponding median (50 percentile) values for each survivor group were determined to be (+)0.245, (–)0.810, and (–)1.009, respectively. Finally, since there was an overlap between the three regions, the author calculated the 25 percentile values (approximated as the half median values) of the high-risk (+0.122) and low-risk (−0.505). Accordingly, these values were used as fixed thresholds for stratifying patient into low-risk (Rad-score < –0.505) for long-survivors (> 15 months) group, medium-risk (Rad-score between −0.505 and 0.122) for medium-survivors (10–15 months) group, and high-risk (Rad-score > 0.122) for short-survivors (<10 months) group.

The mpMRI radiomics signature model was constructed on the discovery data set. Its statistical performance with survival association was assessed in the discovery and validation sets using the *t*-test. True positive rate (sensitivity) and the false positive rate (1—specificity) metrics were used to evaluate the signature model's classification performance in both data sets. The association between the LASSO selected radiomics features and survival in the discovery and validation data sets was illustrated via a heat map, in which the selected radiomics features were rescaled by the z-score transformation.

### Training and Validating a ML Classifier

Several machine learning classification algorithms were assessed in this study for patients' stratification based on survival. The classifiers were trained, and the top-ranked ones reported. Eight various models were included here, and they are listed below:

- *Support Vector Machine classifiers* (Vapnik, 1982):
  1. *Linear SVM*: makes a basic linear separation of classes;
  2. *Medium Gaussian SVM*: creates moderate distinctions between classes, with a kernel scale set to the square root of (P) where P is the number of features/predictors;
  3. *Coarse Gaussian SVM*: creates coarse distinctions between classes, with kernel scale set to the square root of (P) × 4,
  4. *K-Nearest Neighbors (KNN) classifiers* (Patrick and Fischer, 1970):
  5. *Coarse KNN*: creates rough distinctions between classes with the number of neighbors set to 100;
  6. *Cosine KNN*: creates moderate distinctions between classes using a cosine distance metric with the number of neighbors set to 10;
  7. *Medium KNN*: creates moderate distinctions between classes with the number of neighbors set to 10,
  8. *Discriminant Analysis* (McLachlan, 2004):
  9. *Linear Discriminant*: creates linear boundaries between classes, and
  10. *Ensemble Learning*: (Ho, 1998; McLachlan, 2004):
  11. *Subspace Discriminant*: Subspace, with Discriminant Analysis, has medium flexibility and good for many predictors with a few hundred learners. Learning rate set to 0.1 is a popular choice for shrinkage.

All classifiers were trained on the combined data set (*n* = 163). They were trained using various feature combinations: (a) the all radiomics (*n* = 147) features, (b) the LASSO selected (*n* = 9) features, (c) and (d) both features combined with the clinical factors (predictors), respectively. The target response for each model was the patients' OS grouped into three classes representing short- (<10 months), medium- (10–15 months), and long-survivors (>15 months).

A cross-validation scheme with 5-fold (to avoid overfitting) was employed to examine the predictive accuracy of the trained ML classification models and help in determining the best model. The method is commonly recommended for a small data set, as in the case of this study (163 observations). The receiving operating characteristics (ROC) curve was used to check model performance after training each classifier. ROC plot, illustrating the performance of the classifier, displays values of the true-positive and false-positive rates for the model under study. The area under the ROC curve (AUC) was used to measure the performance of individual survival group predicted by a classier, and the accuracy metric to evaluate the overall classifier performance in predicting the three groups. Also, the individual classifier's performance as a function of feature choice was assessed to examine its impact on accuracy.

### The Proposed Method

**Figure 3:**
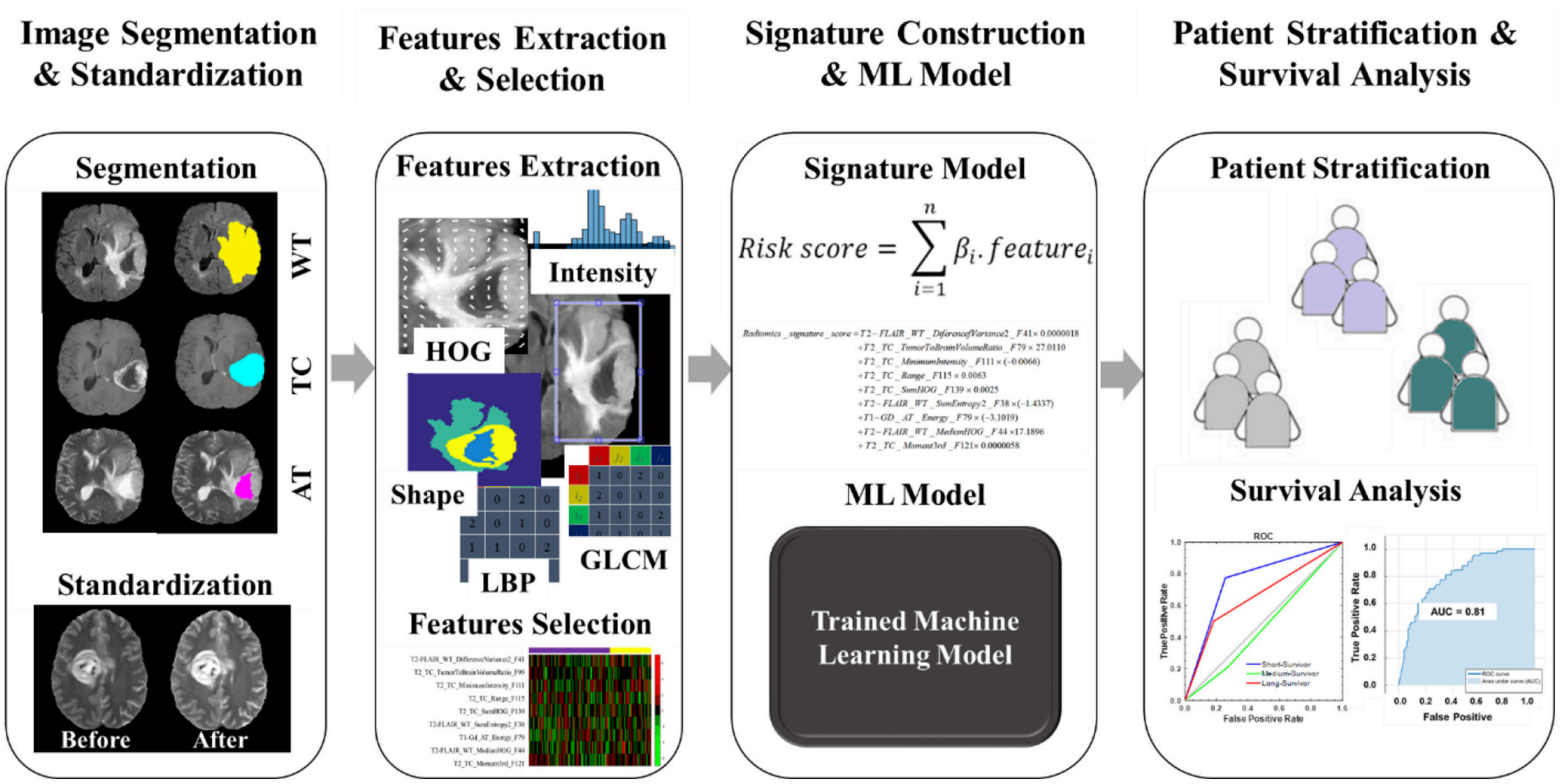
The workflow of radiomics analysis used in this study. The overall procedure of identifying a mpMRI radiomics signature model and a practical ML model for stratifying the GBM patient's prognoses based on overall survival.

The flowchart of the proposed model/method presented in this study for survival prognosis for patients with glioblastoma is demonstrated in Figure 3. It composed of four blocks. Block one for image acquisition, segmentation, and preprocessing, block two for features extraction and selection, block three for signature construction and ML models, and finally block four for patient stratification and survival analysis.

**Figure 4:**
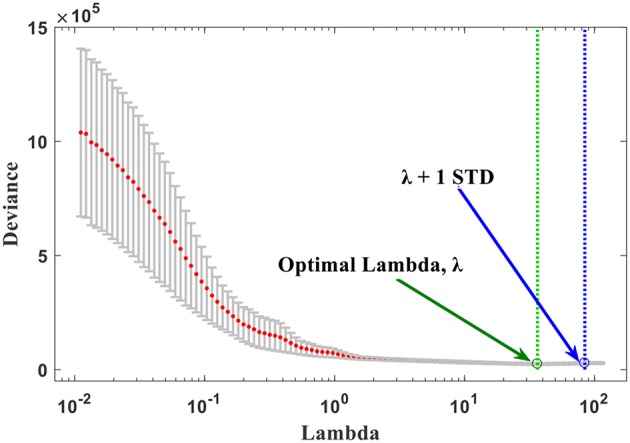
The optimal λ selection by cross-validated deviance of LASSO fit. The partial likelihood deviance plotted vs. λ. The green dotted vertical line was plotted at the optimal λ (36.50) and the blue dotted at λ + 1 STD (84.33) as shown in the plot.

The overall procedure could be summarized as follows: At first, pre-operative multi-parametric MRI (T1, T1-Gd, T2, and T2-FLAIR) sequences are acquired for patients with glioblastoma multiforme (Figure 1). Tumor sub-structures (“whole tumor”, tumor core, and active tumor) are delineated on the acquired images after registering the images with its corresponding reference one. Then the mpMRI intensities are rescaled with a standardized normalization scheme of μ ± 3σ with 256 intensity bins (Figure 2). Secondly, features extraction and selection take place here. Geometry/shape, intensity, HOG, LPB, and GLCM features (Table 2) are derived from the standardized intensity MRIs. Important features with the most relevance to patient survival are selected with LASSO (Table 3 and Figure 4). Thirdly, multivariate LASSO Cox is applied to the selected features to extract the corresponding coefficients. These coefficients are linearly combined to construct a radiomics signature model via risk score. Then, fixed thresholds determined during the signature construction, are used for stratifying patients into a low-risk (Rad-score < −0.505) for long-survivors (>15 months) group, a medium-risk (Rad-score between −0.505 and 0.122) for medium-survivors (10–15 months) group, and a high-risk (Rad-score > 0.122) for short-survivors (<10 months) group. A multivariate ensemble (subspace Discriminant) machine learning model, trained and cross-validated, is used as a more practical model for survival class prediction. And fourthly, using the signature and ML models, glioblastoma individual patients are stratified into short-, medium-, or long-survivors.

**Table 3:** The subset of nine imaging features selected by the LASSO model and the clinical factors with their median, non-zero coefficients determined with Cox regression, and *P*-value for constructing the mpMRI radiomics signature in the discovery data set.

**Characteristics**	**Median**	**Coefficients**	***P-*value**
**Imaging features (LASSO Futures)**			
T2-FLAIR_WT_DifferenceVariance2_F41	132670	1.8000e-06	9.7500e-04
T2_TC_TumorToBrainVolumeRation_F79	0.0057	27.0110	0.0028
T2_TC_MinimumTumorIntensity_F111	123.9908	−0.0066	0.0030
T2_TC_Range_F115	121.5823	0.0063	0.0030
T2_TC_SumHOG _F139	244.4848	0.0025	0.0040
T2-FLAIR_WT_ SumEntropy2_F38	1.9066	−1.4337	0.0152
T1-GD_AT_Energy_F_79	0.2027	−3.1019	0.0175
T2-FLAIR_WT_MedianHOG _F44	0.1107	17.1896	0.0185
T2_TC_Moment3rd_F121	−5324.8	5.8300e-06	0.0203
**Clinical factors**			
Age (years)	61.17	–	3.3700e-04
Resection status (GTR, STR, NA)	–	–	0.9720

*They were ordered by their association with survival (P-value)*.

### Statistical Analysis

All of the statistical data analysis and modeling in this study were performed with MATLAB 2016b software with implemented Statistics and Machine Learning Toolbox (MathWorks, Natick, MA, USA). The differences in patient age, tumor resection status, and OS between the discovery and the validation data sets were evaluated using an independent sample *t*-test (two-sample *t*-test).

## Results

### Clinical Characteristics

The median and mean of overall survival were 362 days and 421 days for the discovery/training data set. For the validation data set, the values were 364 days and 426 days, respectively. The median and mean of age were 60 years and 61 years, respectively, for the discovery data set, and the values for both, median and mean, were 62 years for the validation data set. There was no indication of significant difference in clinical and follow-up data between the discovery and validation data sets (*P* = 0.368 for age test, *P* = 0.474 for tumor resection status test, and *P* = 0.934 for OS test).

### The Radiomics Signature Results

The nine features, selected by the LASSO with non-zero coefficients, formed of 2 from T2-FLAIR, 1 from T1-Gd, and 6 from T2 MRI. These imaging features, plus the clinical factors, are provided in Table 3, arranged in order from high to low importance (*P*-value), with their median, *P*-values, and LASSO Cox regression model coefficients. Each feature was named as Modality_Region_FeatureName_FeatureNumber. For instance, T2_TC_SumHOG_F139 indicated that this feature is the sum of HOG extracted from the tumor core region on T2 MRI sequence and was the feature number 139 in the full list. The optimal λ obtained during the cross-validation of features selection in LASSO regression model was 36.50 with λ + 1 standard deviation (STD) of 84.33 (66.67% confidence level), as shown in Figure 4. As a result, this optimized value, obtained through the cross-validation, has selected nine features with non-zero coeffcients. Usually, as the lambda value increases, the number of non-zero components of predictor coefficients decreases.

Features indicated strong association with survival (*P* < 0.05) from most to least, according to their *P*-value as shown in Table 3 are: GLCM difference variance2 (difference variance calculated at 0 degree and 45 degree rotations) in the WT [T2-FLAIR], tumor to brain volume ratio in TC [T2], minimum intensity in the tumor in TC [T2], intensity range within the tumor in TC [T2], sum of HOG in TC [T2], sum of entropy2 (sum entropy calculated at 0 degree and 45 degree rotations) in WT [T2-FLAIR], GLCM energy in the AT [T1-GD], median HOG in the WT [T2-FLAIR], and momentum 3rd order in the TC [T2].

The linear combination of those LASSO selected nine features enables constructing the radiomics signature. Hence, the signature score (risk score) can be calculated as follows:

$$\begin{array}{l} \;Radiomics\_\\ signature\_score=T2-FLAIR\_WT\_DifferenceVariance2\_F41\\ \;\times 0.0000018\\ \;+T2\_TC\_TumorToBrainVolumeRatio\_F79\\ \;\times 27.0110\\ \;+T2\_TC\_MinimumIntensity\_F111\times (-0.0066)\\ \;+T2\_TC\_Range\_F115\times 0.0063\\ \;+T2\_TC\_SumHOG\_F139\times 0.0025\\ \;+T2-FLAIR\_WT\_SumEntropy2\_F38\times (-1.4337)\\ \;+T1-GD\_AT\_Energy\_F79\times (-3.1019)\\ \;+T2-FLAIR\_WT\_MedianHOG\_F44\times 17.1896\\ \;+T2\_TC\_Moment3rd\_F121\;\times 0.0000058 \end{array}$$

When the radiomics score value has been determined through the above-given signature model, the glioblastoma patient can be stratified accordingly into one of the survival groups. The thresholds, established with the ideal cutoff points on the discovery set, were low-risk (Rad-score < −0.505) for long-survivors (>15 months) group, medium-risk (Rad-score between −0.505 and 0.122) for medium-survivors (10–15 months) group, and high-risk (Rad-score > 0.122) for short-survivors (<10 months) group.

**Figure 5:**
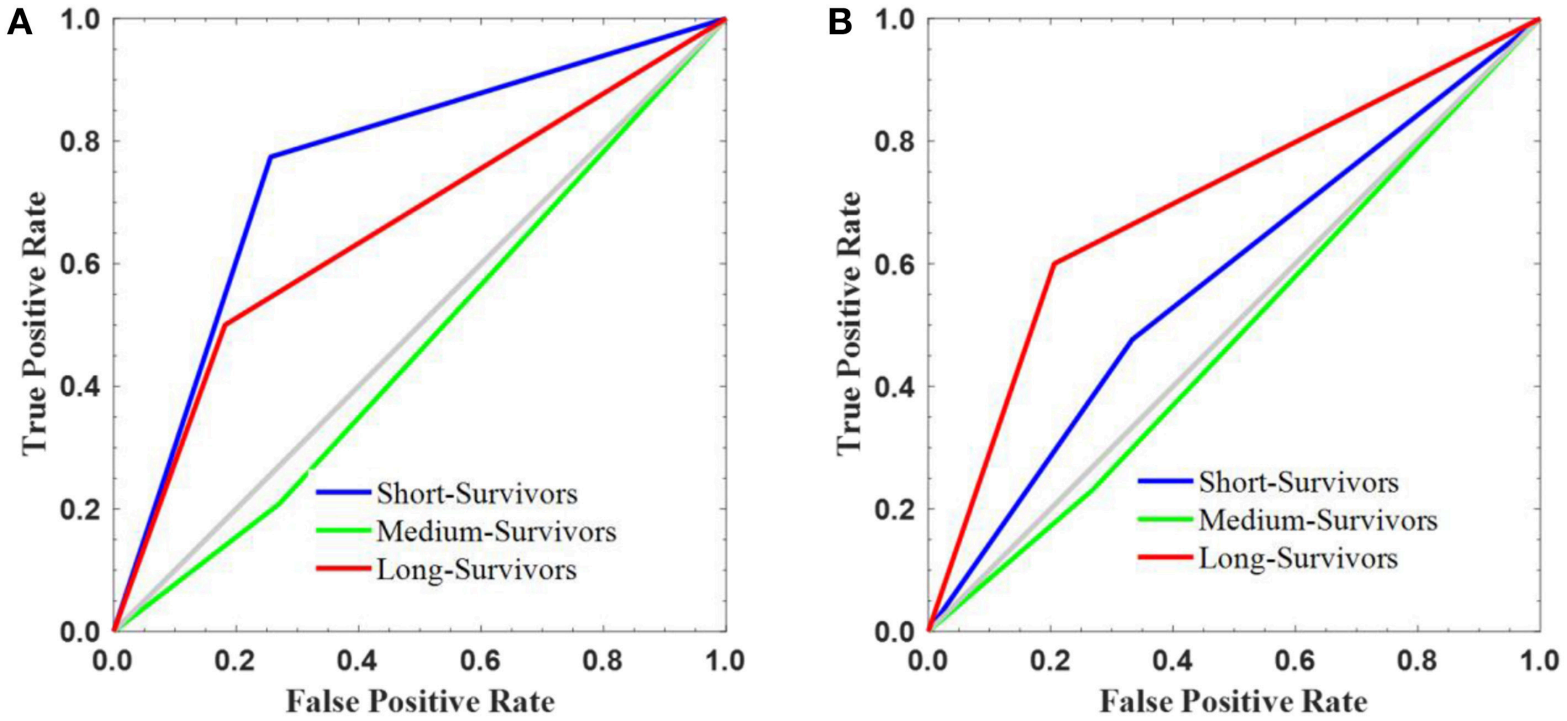
The survival stratification created using the constructed radiomics signature. The signature performance in stratifying the survival into short-, medium-, and long-survivors on the **(A)** discovery and **(B)** validation sets.

The signature model performance in both, discovery and validation, data sets stratified the patients according to the pre-determined fixed criteria/cutoff points were shown in Figure 5. A significant association (*P* < 0.001) of the radiomics signature with OS was shown in the discovery data set, but non-significant correlation (*P* = 0.110) was observed in the validation data set.

On discovery cohort, the radiomics signature stratified the GBM patients based on survival grouping with the true positive rate or sensitivity metric as following: short- (0.774), medium- (0.208), and long-survivors (0.500). The false positive rate (1—specificity) measure was 0.256, 0.271, and 0.182 for short-, medium-, and long-survivors, respectively (Figure 5A). In contrast, the reported values on the validation set were 0.476 (short-), 0.231 (medium-), and 0.600 (long-survivors) for true positive rate or sensitivity; and 0.333 (short-), 0.268 (medium-), and 0.206 (long-survivors) for false positive rate (1—specificity) (Figure 5B). For example, a false positive rate of 0.256 demonstrates that the signature model on the discovery data set assigns 26.8% of the long-survivors predictions falsely to the positive class. On the other hand, a true positive rate of 0.600 points out that the signature model classifies 60% of the predictions correctly to the positive class.

**Figure 6:**
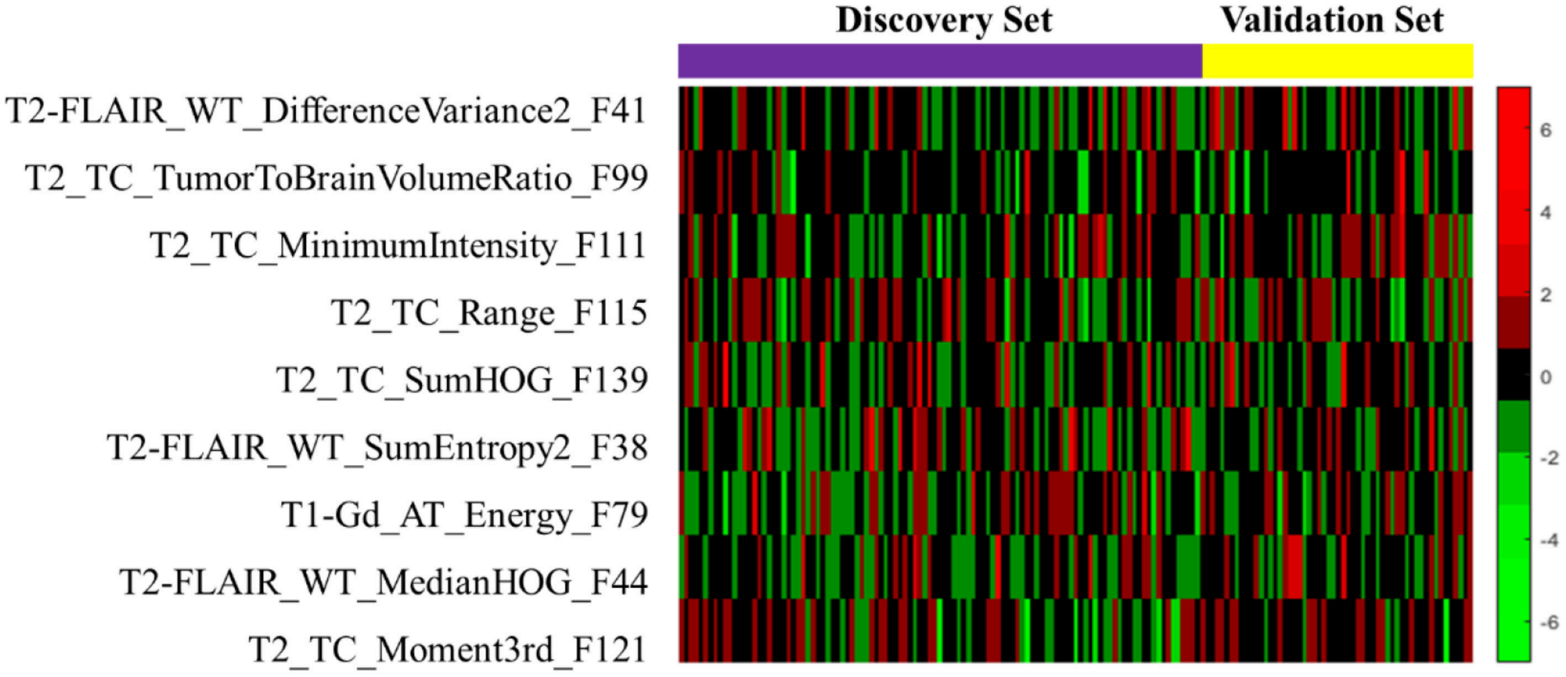
The heat map of the LASSO selected radiomics features that used to discover the signature. The rows demonstrate the subset of nine selected features, while the columns indicate the patients (both discovery and validation data sets). The color map shows the z-score difference of each radiomics feature.

The heat map of the 9 LASSO selected features used for building the signature is shown in Figure 6. It shows the features association with OS between the discovery and validation data sets. From the heat map plot, it can be noticed that there is a consistency of radiomics feature z-score between the discovery/training and the validation data sets.

### ML Model Results

Eight machine learning classification models were examined for survival prediction, and their performances were presented in Table 4. The AUC for predicting an individual survival class from the other classes, and the overall accuracy results, are reported for each model. The overall best model with feature combination for classifying OS into three groups was identified.

**Table 4:** AUC and overall accuracy of several trained ML models' performance in classifying GBM patients survival into three groups as a function of choice of features.

**Classifiers and features**	**AUC**			**Overall accuracy (%)**
	**Short-survivors**	**Medium-survivors**	**Long-survivors**	
**SVM (Medium Gaussian)**				
•Imaging features	0.67 (0.69)	0.52 (0.60)	0.61 (0.59)	47.2 (50.3)^*^
•Imaging features + clinical factors	0.74	0.51	0.67	53.4
•Imaging features (LASSO)	0.72 (0.74)	0.31 (0.37)	0.68 (0.73)	50.9 (56.4)^**^
•Imaging features (LASSO) + clinical factors	0.80 (0.81)	0.51 (0.53)	0.68 (0.73)	54.0 (55.2)^**^
**K-Nearest Neighbors (Coarse KNN)**				
•Imaging features	0.64	0.48	0.60	46.0
•Imaging features + clinical factors	0.68	0.46	0.67	50.1
•Imaging (LASSO) features	0.73 (0.72)	0.47 (0.45)	0.72 (0.67)	47.2 (50.3)^†^
•Imaging (LASSO) features + clinical factors	0.79 (0.78)	0.44 (0.55)	0.70 (0.66)	47.9 (50.9)^††^
**Discriminant analysis (Linear)**				
•Imaging features	0.67	0.52	0.61	47.2
•imaging features + clinical factors	0.72	0.48	0.67	49.1
•Imaging (LASSO) features	0.74	0.45	0.72	56.4
•Imaging (LASSO) features + clinical factors	0.79	0.49	0.71	53.4
**Ensemble (Random subspace discriminant)**				
•Imaging (LASSO) features	0.75	0.42	0.71	57.1
•Imaging (LASSO) features + clinical factors	**0.81**	**0.47**	**0.72**	**57.8**

* *Values in brackets are the performance of SVM Linear classifier*.

** *Values in brackets are the performance of SVM Coarse Gaussian classifier*.

† *Values in brackets are the performance of KNN Cosine classifier*.

†† *Values in brackets are the performance of KNN Medium classifier*.

*The overall best classification results are listed in bold*.

The best overall performance classifier was achieved by an ensemble learning model with AUC of 0.81, 0.47, and 0.72 for short-, medium-, and long-survivors (Table 4), respectively. The corresponding overall accuracy was 57.8% in predicting the patient's survival into short-, medium-, and long-survivors group. Combining the LASSO selected imaging features with the clinical predictors yielded in improved prediction accuracy results over the other alternatives in estimating glioblastoma patients' survival.

**Figure 7:**
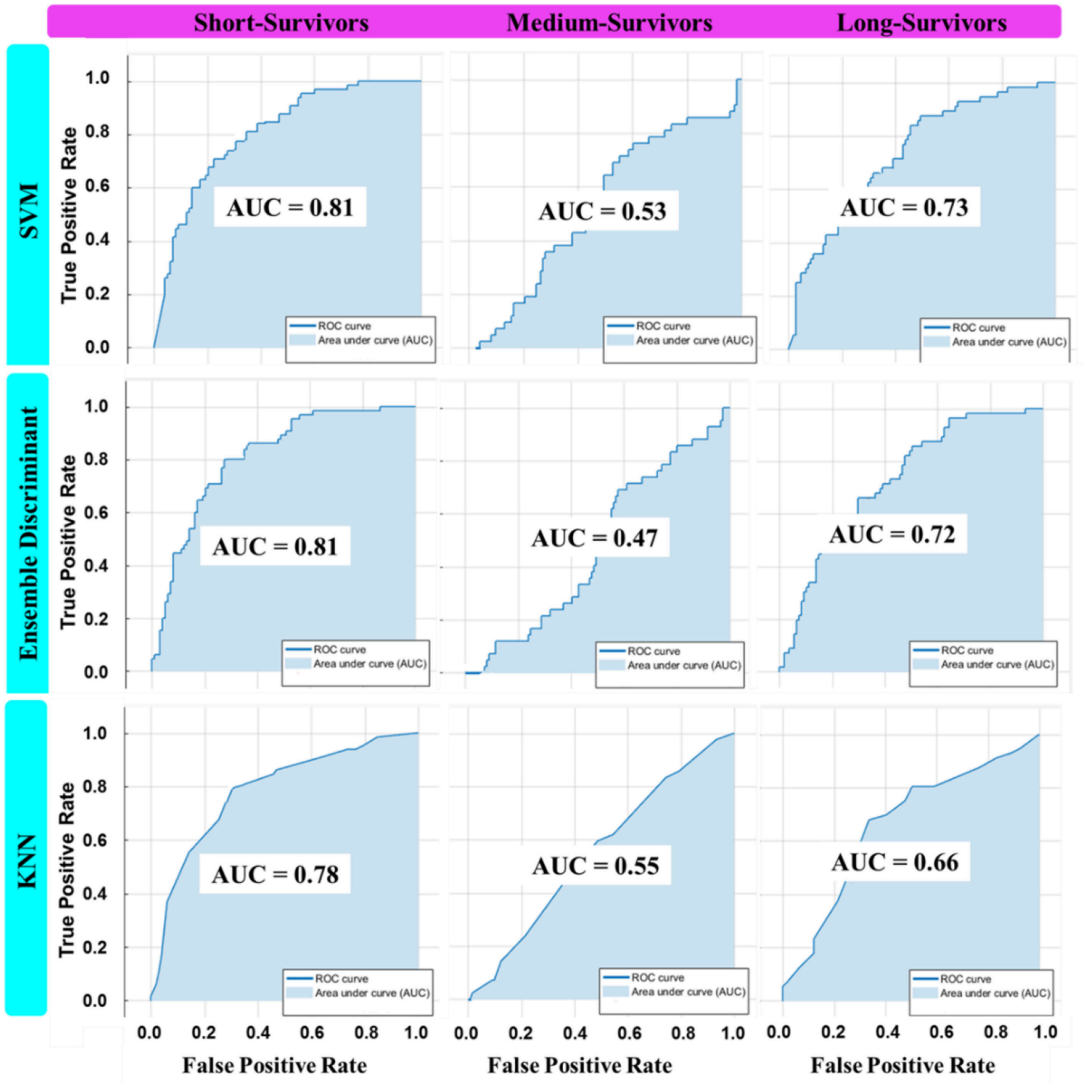
The AUC plot of the three best overall ML classifier invariants in each machine learning category: SVM (Coarse Gaussian), KNN (Medium), and Ensemble (Subspace Discriminant) in classifying OS into three classes using the best feature combination.

The AUC plots of the three classification models, including the ensemble model (the superior one among the other alternative models), were shown in Figure 7. Ideally, the perfect AUC plot is a right angle to the top left of the plot (with no misclassified points). The AUC value measures/quantifies the overall quality of the classification model. The larger AUC value demonstrates better model performance. Figure 7 shows the AUC values for each survival class/group individually. In other words, it quantifies how the model under study is capable to classify a specific group of survivors from the other classes correctly.

### Results Comparison

A comparison of this study results with other published works was presented in Table 5. The proposed model performance, the signature plus the ML model, was judged amongst other works in various manners.

**Table 5:** The comparison of this study's findings with similarly published works for GBM patients stratification based on survival with radiomics analysis.

**Method**	**MRI sequences**	**Feature selection and classification models**	**Survival stratification**	**Overall accuracy**	**AUC**	**Signature model association with OS**
Yang et al. (2015)	T1 and T2-FLAIR	Ensemble (random forest) learning	12-months survival	–	0.67	–
Macyszyn et al. (2016)	T1, T1-Gd, T2, T2-FLAIR, DTI, and DSC	SVMs	Short- (<6 months), medium- (6–18 months), and long-term (>18 months)	80.0%	–	–
This work	T1, T1-Gd, T2, and T2-FLAIR	LASSO and Cox regression, ensemble (subspace discriminant) learning	Short- (<10 months), medium- (10–15 months), and long-term (>15 months)	57.8%	0.81, 0.47, 0.72	Discovery (*P* < 0.001), validation (*P* = 0.110)
Sanghani et al. (2018)	T1, T1-Gd, T2, and T2-FLAIR	SVMs	Short- (<10 months), medium- (10–15 months), and long-term (>15 months)	88.95%	–	–
Liu et al. (2018b)	T1, T1-Gd, T2, and T2-FLAIR	SVMs	Short- (<12 months) vs. long-term (≥12 months)	80.7%	0.79	–
Chen et al. (2019)	T1-Gd	LASSO Cox regression	Short- (<12 months) vs. long-term (≥12 months)	85.1%	0.81	Discovery (*P* < 0.001), validation (*P* < 0.001)
Chaddad et al. (2019b)	T1-Gd and T2-FLAIR	Random forest	Short- (<12 months) vs. long-term (>12 months)	–	0.78	–
Zong et al. (2019)	T1, T1-Gd, T2, and T2-FLAIR	CNNs	Short- (<6 months), medium- (6–18 months), and long-term (>18 months)	64.3%,	–	–
Rathore et al. (2019)	T1, T1-Gd, T2, T2-FLAIR, DSC-MRI, and DTI	K-means clustering, Cox regression	Worst (MS = 6 months), intermediate (MS = 12 months), and longest survival (MS = 19 months)	–	–	Validation (*P* < 0.001)

*DTI, Diffusion Tensor Imaging; DSC, Dynamic Susceptibility Contrast-Enhanced; CNNs, Convolutional Neural Networks; MS, Median Survival*.

## Discussion

Radiomics analysis is the concept of extracting features quantitatively from the images/medical images using a variety of computational approaches. Then, the obtained imaging features may be used to provide clinicians with diagnosis, prognosis (e.g., survival), or treatment response. This study was aimed to identify a radiomics-based imaging signature on pre-operative mpMRI to stratify patients with *de novo* glioblastoma multiforme into short-, medium-, and long-survivors group using data from multiple institutions. Also, establishing a practical ML model for the same purpose through testing a wide range of various classification models and different features combination. Statistics, Computer Vision, and Machine Learning tools were used implementing the proposed model of radiomics analysis of patient stratification based survival grouping, which may offer unique clinical insights to support decision-making toward precision oncology.

Various image features (*n* = 147), representing tumor's shape, intensity, GLCM, HOG, and LBP (Table 2), were extracted and derived via different approaches on multi-parametric MRI (T1-Gd, T2, and T2-FLAIR) sequences characterizing the tumor structures [AT, TC, and WT (Figure 1)]. When a two-step feature selection method was employed, MAD followed by LASSO regression (Figure 4), a final set of 9 features retained (Table 3). LASSO turns all none relevant features/variables coefficients to zero during the optimization and tunes the regression model via a user-specified k–fold cross-validation. It performs both feature selection and regularization to improve the prediction accuracy and the interpretability of the statistical model it produces. The selected features indicated a high association with OS (*P* < 0.05) as shown in Table 3. Among those features, a gray-level co-occurrence matrix derived texture feature has shown the highest association with GBM survival stratification (Table 3). This finding agrees with that reported in the literature (Chaddad et al., 2015, 2016a). Image entropy and energy selected features have also shown a good correlation with survival (Chaddad et al., 2016b, 2019b; McGarry et al., 2016). Those features, typically calculated within a region of interest, indicate that intra-tumoral heterogeneity has a high impact on the survival stratification. The quantitative nature of radiomics features and the qualitative nature of radiologists to interpret the MRI sequences could complementary improve the GBM patient survival prognosis quality toward precision oncology.

A multi-parametric MRI radiomics signature of 9 features was constructed on the discovery cohort for glioblastoma patients stratification based on overall survival. LASSO Cox regression model was used to extract the selected features' coefficients (Table 3) for developing a signature model. The author discussed the reason for applying this approach in the method section. Also, it has been reported that regression coefficients estimated by the LASSO are biased by intention, but can have smaller mean squared error than conventional estimates (Heinze et al., 2018). The radiomics signature model, trained and validated, had a good performance (*P* < 0.001) with survival association in the discovery set (n = 109), but this results not confirmed (*P* = 0.110) in the validation set (*n* = 54) (Figure 5). The possible reasons for non-significant results obtained in the validation set could be due to signature model overfitting during the training. It has been reported that over-fitting is possible when the number of features is greater than the number of data samples or if there are too many unique values for a discrete feature (Meinshausen and Bühlmann, 2006). The poor results obtained show the lack of generalizability of the signature model on the new unseen data set. From the statistical perspective, non-significant relationship with survival does not necessarily mean less importance (Lao et al., 2017). A second reason could be due to high contribution (almost a half, 49% as shown in Table 1) of patient data with missing resection status information in the combined, discovery/training and validation, cohort. These data with unknown resection information could significantly affect the overall or/and individual, training or validation, results. And, a third reason could be due to possible sub-optimal determination the cutoff points' values or thresholding in which some possibly valid assumptions had applied.

The machine learning results of several studied classifiers indicated the superiority of ensemble (Subspace Discriminant) learning over the other methods achieving the best performance accuracy of 57.8% (Table 4 and Figure 7) in categorizing the survival into short-, medium-, and long-survivors. This result is not sufficiently encouraging and more tuning is needed for improved prediction accuracy. The LASSO selected imaging features, combined with clinical factors, provided better prediction results among the other options. According to the survival data distributions used in this study (Table 1), the best survival grouping achieved for predicting short-survivors (representing 40% of the total OS data distribution) with an AUC of 0.81. Then it followed by long-survivors (representing 36% of the total OS data distribution) with an AUC of 0.72. Finally, medium-survivors (representing 26% of the total OS data distribution) were lasted with an AUC of 0.47. Lower performance in predicting an individual class correlated with a decreased class data distribution in the study sample. Strengths and limitations of the ML classifiers used in this study could be summarized here. Based on prediction speed, all reported models were relatively fast. In contrast, Linear models (SVM and Discriminant Analysis) are easy to interpret, SMV (with Gaussian kernels, Medium, and Coarse), KNN (Coarse, Cosine, Medium), and Ensemble (Subspace Discriminant) are hardly interpretable.

The results comparison of the proposed method (signature model and the practical ML model) with most relevant published studies are presented in Table 5. While the proposed method's results, the signature model and the ML model, was not impressive compared to most recently reported works (Macyszyn et al., 2016; Liu et al., 2018b; Sanghani et al., 2018; Chen et al., 2019), it was comparable or even better with respect to others studies for example that reported by Yang et al. (2015) (AUC = 0.67 for 12 months survival prediction) and Chaddad et al. (2019b) (AUC = 0.78 for short- vs. long-term OS prediction). Also, this study results are relatively comparable with that obtained by Zong et al. (2019) on multi-institutional data (accuracy of 64.3% for three-class OS prediction) using Convolutional Neural Networks, where CNN based methods are commonly expected to provide much-improved performance compared to traditional methods. The works by Macyszyn et al. (2016), and Rathore et al. (2019), reported good performance results in predicting GBM patient's survival group. However, these studies were conducted on a single institution's data, where the data is more homogeneous/consistent and more likely to obtain improved accuracy than the one used multiple institutions as a case in this study. Consequently, the model trained in local data is likely to suffer in generalizing its performance to unseen data from other institutions. On the other hand, a model trained on multi-institution data may gain generalizability but less prediction accuracy due to the heterogeneity of the data.

Finally, this study establishes that multi-parametric MR images in patients with glioblastoma hold prognostic information, which can be called up by radiomics analysis via Statistics and Machine Learning/Computer Vision methods. The proposed method in this study still has some limitations and weaknesses, which may have influenced its reported results. This work represents a retrospective study from multiple institutions with a relatively small sample patient data set used on discovery (*n* = 109) with an independent validation data set (*n* = 54) for signature model construction and evaluation. Also, almost half (49%) of the clinical data information/predictors were with no given tumor resection status (GTR or STR) information (Table 1). By making available a large standard multi-institution data set, it would enable us to fully evaluate the generalizability, and thus improve the performance of the radiomics signature model on the new unseen data set.

## Conclusions

Image features were extracted from pre-operative multi-parametric MR images of patients with glioblastoma to generate a radiomics signature model and a practical ML model for stratifying patients into groups based on overall survival. A derived gray-level co-occurrence matrix feature was found to have a high association with survival, which means that intra-tumoral heterogeneity has an essential role in the survival stratification. The proposed radiomics signature model had good performance in the discovery set and lower performance in the validation cohort. Despite the limitations, the offered signature model has the potential for improved pre-operative care of glioblastoma patients. Ensemble learning showed superior performance over the tested ML classifiers for survival prediction as a function of the choice of features. Clinical factors, when added to the radiomics imaging-based features, boosted the performance of the machine learning classification model in predicting individual glioblastoma patient's survival prognosis. These findings may help in choosing an optimal treatment strategy and assist in making personalized therapy decisions of glioblastoma patients which improve prognostic quality and represent a step forward toward precision oncology.

## Data Availability

Publicly available data sets analyzed for this study. The data sets can be found in the BRATS 2018 challenge (https://www.med.upenn.edu/sbia/brats2018/data.html).

## Author Contributions

AO conceptualized and designed the study, developed the models, performed the statistical analysis and interpretation, created a computer graphics visualization of the results, wrote the first draft and the sections of the manuscript, and revised and approved the submitted version.

### Conflict of Interest Statement

The author declares that the research was conducted in the absence of any commercial or financial relationships that could be construed as a potential conflict of interest.
